# Happy first birthday, *PNAS Nexus*

**DOI:** 10.1093/pnasnexus/pgad020

**Published:** 2023-03-03

**Affiliations:** *PNAS Nexus*, USC Viterbi School of Engineering, University of Southern California, Los Angeles, CA 90089-1450, USA

March 2023 will be the first anniversary of the publication of *PNAS Nexus*. Launched in August 2021 as the only other research journal of the National Academy of Sciences since the founding of PNAS in 1914, *PNAS Nexus* is its precocious sibling, which benefits from their common lineage. Indeed, in the short time since its founding, the new journal has received more than 1,200 submissions. *PNAS Nexus* reflects the convergence of disciplines across the sciences, engineering, and medicine that we vividly witness today, and projects the incredible intellectual breadth and depth of the three national academies. Complementary to PNAS in scope and focus, *PNAS Nexus* thrives on publishing innovative, interdisciplinary work from researchers worldwide.

**Figure:**
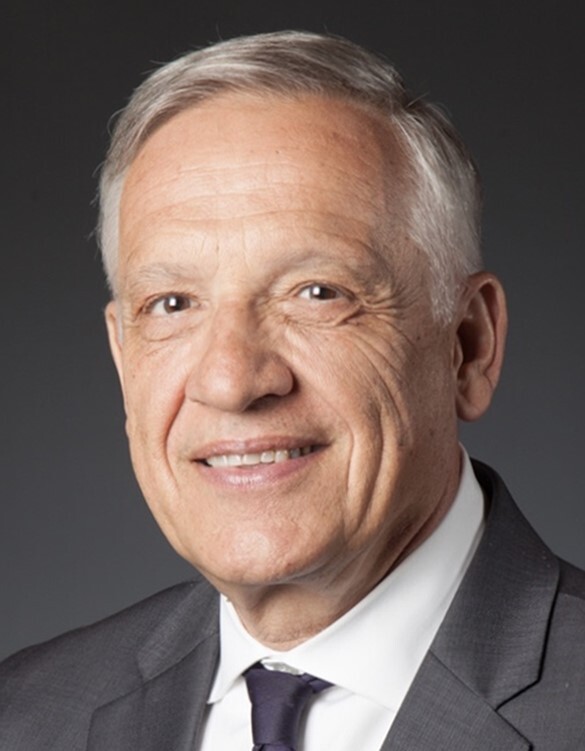
Yannis C. Yortsos

We live in extraordinary times. To quote the late National Academy of Engineering President Chuck Vest, “This is the most exciting time for science and engineering in human history.” The exponential rate of change in scientific discovery and technology brings unprecedented opportunities for successfully addressing grand, challenge-like problems: sustainability, health, security, and enriching life; from scientific discovery to societal organization to innovation.^1^ But at the same time, this empowering technological prowess is the root of equally powerful unintended consequences, almost all of which are driven by human nature.

*PNAS Nexus* lives at these fertile intersections: between physical and biological sciences, engineering and technology, social sciences, and unintended consequences. The convergence of disciplines will continue to intensify in the years ahead, particularly as the interface between technology and humanity becomes increasingly intertwined, bringing important, perhaps existential, questions to the fore about what it means to be human or how we can secure a sustainable future for the generations that will follow us.

A surrogate word for convergence is interdisciplinarity. It is perhaps an apt description, although it also echoes some concepts that may be outdated. Indeed, because of the extraordinary speed of scientific and technological innovation, almost every scientific discovery has a nearly immediate impact across many disciplines. Think AI. More importantly, impacts on society are direct and potentially immense—although not easily predicted, partly because many of them come about as a result of unintended consequences. *PNAS Nexus*, being intentionally interdisciplinary, embodies such mindsets for discovery and impact, which are in a way its raison d’être.

Here is a bit of my thinking about the various terms used to describe the journal, from multidisciplinary to interdisciplinary and transdisciplinary:

- Multidisciplinary implies the publication of manuscripts that collectively cover a number of different disciplines.
- Interdisciplinary implies manuscripts that address the intersection of one or more disciplines authored by collaborators from more than one discipline.
- Transdisciplinary implies manuscripts that create new scientific domains, likely combining two or more disciplines—for example, Imageomics, a new field of science at the crossroads of biology and machine learning.

Regardless of their specific nature, their *PNAS Nexus* attribute is that they should provide outstanding new advances, with novelty and excellence and a strong promise of longevity to withstand the test of time.

As the editor-in-chief since late November 2022, I fully realize this position's honor, privilege, challenges, and heavy responsibility. The Academies’ trust in me to help implement the vision of *PNAS Nexus* is gratifying and inspiring. The opportunity is in front of us to make *PNAS Nexus*—the only sibling to PNAS—an exceptional journal for the sciences, engineering, and medicine and their common convergence. And for it to become a premier worldwide journal that spans and enables disciplines—a journal ideally suited for our times.

## Footnotes

1 See http://www.engineeringchallenges.org/challenges.aspx for more information about the NAE Grand Challenges for Engineering.

